# The Medical Treatment of Insanity

**Published:** 1857-09

**Authors:** M. H. Ranney

**Affiliations:** Resident Physician of the New York City Lunatic Asylum, Blackwell’s Island


*The Medical Treatment of Insanity.* By M. H. Ranney, M. D., Resident Physician of the New York City Lunatic Asylum, Blackwell's Island.

*Read before the Association of Medical Superintendents of American Institutions for the Insane, May,* 1857.

In presenting my views relative to the medical treatment of Insanity, I shall be very brief, tracing only the general outlines of the course pursued by me in a few of the best-marked forms of this disease.

The object of the report is to obtain an expression of the views of the different members of the Association on this subject, to attain which it is necessary that there be no misunderstanding as to the particular disease described. The treatment of insanity is the great desideratum, although in fact subjects of secondary importance are much more frequently discussed. The peculiar ideas entertained by the members as to ventillation, the construction of water-closets, &c., are generally understood; but I

am unable to say that there is an unanimity of opinion as to the mode of practice in any one of the various forms of insanity. It may be, perhaps, impossible to determine the exact treatment which should be pursued in a particular case, but the general principles, at least, that govern our course in a certain defined form of mental derangement, can be given as well as in the treatment of physical diseases generally. I assume that there are conventional terms, which convey to the mind definite ideas of certain forms of disease which, when referred to, suggest a group of associated symptoms that, taken collectively, constitute a distinct variety. It is only to a few of such well-known and recognized forms that reference will be made.

In insanity no new faculties are created, but those already existing are modified by the conditions of exaltation, depression, or perversion. The type of the different varieties of disease may be found in the normal state of the mind. This consideration affords important aid in distinguishing one form of mental disease from another.

I shall first refer to Acute Mania. The physiological type of this disease is given more nearly in anger marked by violence than in any other state of mind. The leading characteristics are, impassioned, moral and intellectual exaltation (the one exhibited by perversion, the other by delusion,) the rapid flow of ideas, violent gesticulation, disposition to overthrow or destroy the furniture of the room, sleeplessness, and wild expression of the eye and countenance, betraying great disquiet of mind. Undoubtedly the term of acute mania recalls a certain grouping of symptoms, and conveys more accurate notions of the condition than would the minute description of an individual case, since by abstraction the essentials in particular instances have been selected and combined to form the general idea. Taking it as granted that the form referred to is fully recognized, the medical treatment will be briefly considered. A careful examination must be made into the general condition of the system, as well as of the functional disturbance of any organ that might affect the brain. The success following treatment depends much upon the care exercised in the duty. The patient should, as far as possible, be excluded from all excitement. In most cases the condition of the stomach and bowels are disordered, to correct which an active cathartic should be prescribed. For this purpose the combination, hydrarg. sub. mur. gr. x, pulv. jalap. gr. xx, may be administered, and if the patient be of full habit a grain of tartarized antimony may be added. The skin is often dry and unclean, requiring, after catharsis, a warm bath, and pulv. ipecac. c. gr. x, the following night. On the succeeding day, if the patient be plethoric and there seem to be a determination of blood to the brain, commence with ant. et potass. tart., gr. ss. *ter in die*, which should be gradually increased until nausea follows ; cold applications may be made to the head, and spts. ammon. ascetat., or spts. æth. nit. to act upon the secretions. If there be unnatural rapidity in the pulsations of the heart still persisting, tinct. verat. virid. gtt. v, ad. x, *bis in die*, may be substituted for the tartar emetic. If for several days the patient continue violent, ol. tiglii. is to be applied to the back of the neck and behind the ears; selecting for this a proper time in th: advance of the disease, a full eruption is usually followed by marked improvement. As soon as the prominent symptoms of violence yield, morph. sulp., gr. ss. *ter in die*, is substituted for the remedies before specified, or if, at the time of admission, the patient be emaciated and apparently prostrated, either morphia or opium is given directly after the warm bath. Under these circumstances a full diet is urged, and if with restlessness and high excitement an anæmic state of the brain is believed to exist, a supply of meat rich in fat is liberally furnished. Beer and milk-punch take the place of other drinks. Tonics, such as ferri carb., potass. iodid., &c., have a favorable action, and even quinine is occasionally admissible. When violent paroxysms are separated by lucid intervals, as in recurrent mania, quinine, in doses three times a day during the quiet period, has been found highly beneficial.

My attention was first called to the use of this article by a paper read before this Association three years ago, by Dr. Tyler. Since then I have often used quinine in cases of the recurrent form of insanity with decided success. In many the lucid interval was prolonged, the paroxysm less severe, and in few instances complete recovery was the result. If masterbation was suspected as a cause, free applications of croton oil were made to the penis and scrotum.

Amenorrhœa is a frequent cause of mania in girls between the ages of 15 and 25, while in later life menstrual disturbances usually produce melancholia. Mania from this source yields readily to proper treatment. The tr. al. et myrrh. to remove constipation, Lugol's solution, or some other form of iodine, with stimulating applications to the mammæ, effect, ordinarily, a cure in two or three months. In that form of mania in which little violence exists—the patient seeming like one inebriated, yet moved by that same mischievous propensity that is found in a variety of nymphomania, opium in large doses controls quite effectually the undue exhilaration of spirits. The common course is to commence with tinct. opii., 1 dr. *ter in die*, which is doubled at the expiration of the first, or even increased to three drachms, if found necessary, at the end of the second week. From the peculiar state of the brain and nervous system, these large doses are not only tolerated, but produce little sensible effect aside from allaying the excitement and occassioning active emisis and catharsis. These last conditions render it often necessary to omit the medicine for a day.

Melancholia, the lypemania of Esquirol, is another form of mental disease readily recognized. The elementary type is found in fear, sorrow, or grief, as exhibited by a mother in the loss of her child, or in impending calamity, The peculiar marks which distinguish this affection are exaltation of the sentiment of sorrow, entire concentration of mind on one idea or class of ideas, and an inability to direct the attention to any thing not immediately connected with that which wholly absorbs the mind. It is frequently dependent on some bilious or uterine derangment, and in the selection of medicines attention should be directed to this fact. To correct the secretions mass. hydrarg., or the hydrarg. cum cretâ, may be used. Where a sufficient alterative effect has been produced, opiates in small doses are indicated. The object is to partially remove the intense grief or fear which characterizes this form of disease. Morphia in small doses may for a long time be continued. During its administration gentle laxatives will be required; for, aside from the effect of opiate, there is a tendency to constipation. The patient generally refuses a proper amount of nourishment, leaving the vital powers greatly reduced, and requiring tonics and stimulants—such as ferri carb., porter, *&c.* If a propensity to commit suicide exist, the occasional application of blisters, or ung. antimon. to the back of the neck, lessens much the danger of such an occurrence. It may afford benfit, in part, by relieving congestion of the vessels of the brain, but principally from the substitution of a real for an imaginary trouble.

Of the remaining forms of Insanity Dementia alone is that which I now shall consider. Its fundamental type or analogous exists in natural dullness of intellect. The leading characteristic is an enfeeblement of the intellectual faculties, or even a complete obliteration of their manifestations. Dementia is usually a sequel of mania or some acute affection of the brain; rarely an idiopathic disease. Moral treatment is of much more importance than in mania, or melancholia, yet a judicious use of medicines will aid much in the restoration of reason. To relieve anæmia, nutritious diet and the free use of chalybeates are requisite. The object is to supply the brain its proper stimulous by enriching the blood, and thus arousing its dormant excitability. As the muscle loses its contractile power from long inaction, so may the brain, although uncharged in structure, cease to perform its proper functions, from previous long-continued disease. The phosphates of iron and manganese become valuable in this disease by furnishing the necessary amount of phosphorus for generating the nervous force. In a few instances rapid improvement has followed the use of cannabis indica, which seems to have a special tendency to stimulate the senses, and excite the moral qualities. Those cases in which dullness of intellect depends on a congestive condition of the brain are benefitted by counter irritants, such as blisters, ung. antimon., or ol. tiglii applied to the back of the neck. The most favorable results occasionally follow accidental sloughing from the application of tartarized antim ny, while the same effect may occur from an extensive abscess.

Such are my views in regard to the ordinary course to be pursued in treating the foregoing forms of insanity, each individual case requiring, however, modifications of treatment corresponding to the particular cause, age, sex, temperament, condition of system, &c. Adopting the somatic theory as to the proximate cause of insanity, that the material part, the brain, is the seat of disorder, while the immaterial is not subject to change, there can be no reason why medicines should not exert a controlling influence over this disease. Not only is the physical organization directly affected by medicinal agents, but over the mind itself the manifestation of the immaterial through the medium of the brain is subjected to their restoring influence. Narcotics, especially, seem to act immediately on the brain, producing a marked physical effect. Some excite the senses, others produce in the intellect the most brilliant images, and a few exert their influence over the moral faculties. The first effect of opium is to allay the passions, not only by lessening directly the most violent anger and poignant grief, but also by occupying the attention with fanciful and pleasant imagery, tending to induce cheerfulness and contentment. Hyoscyamus, on the contrary, is supposed to arouse anger and jealousy, while belladonna, in large doses, occasions gloomy thoughts and dejection of mind. Stramonium affects the senses primarily, and, in moderate quantities, disposes to convulsive merriment. From the use of cannabis the activity of the senses is increased, and the most surprising delusions follow, which may continue long after the immediate stimulus has passed away. The effects of narcotics are not more fully understood, but sufficient is known of them to call for a careful discrimination in their use. It is well settled that they act on the mind, and that each has some peculiar characteristic distinguishing its action. If this be granted, it necessarily follows that with a knowledge of the change produced by this class of remedies on the different faculties of the mind, a proper selection for the individual case must be attended with good results.

In thus presenting my views it must not be understood that I advocate entire reliance on medicinal agents in the treatment of insanity. The adoption of proper hygienic rules is essential, as in physical disease generally. Moral treatment, including employment, amusements, the establishment of regular habits, &c., is also a most important auxiliary to recovery. This is particularly true where derangement of mind has existed for years. But while admitting the importance of moral treatment, I would avoid an over-estimate of its mechanical part, and carefully investigate not only the laws of physical action, but the influences of medicine on the manifestations of mind, that our noble profession may not become simply an art.—*American Journal of Insanity.*

